# Supplementary material for: Proteomic analysis of the SMN complex reveals conserved and etiologic connections to the proteostasis network
Source: Front RNA Res. Author manuscript; Available in PMC 2024 Nov 1. (PMC11529804; doi:10.3389/frnar.2024.1448194)
Supplement: Supp Figure S1 [file NIHMS2027894-supplement-Supp_Figure_S1.pdf]

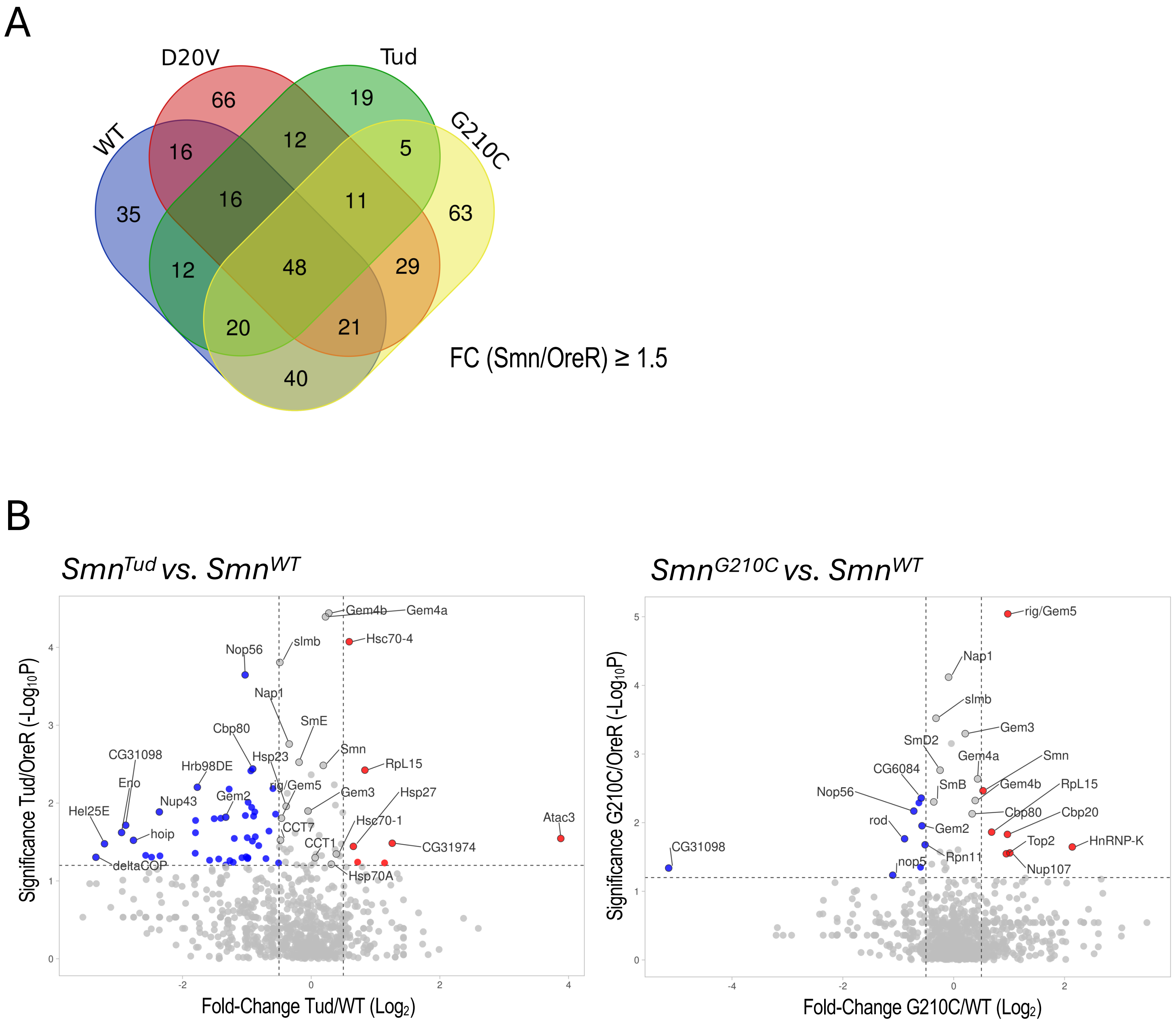

Supplementary Fig S1: A) Venn diagram of proteins enriched (>1.5x) in AP-MS pulldowns of WT, D20V, Tud or G210C constructs. B) Difference plots of shared proteins identified in the Tud vs WT or G210C vs WT AP-MS experiments.

Fig. S1
