# Supplementary material for: Proteomic analysis of the SMN complex reveals conserved and etiologic connections to the proteostasis network
Source: Front RNA Res. Author manuscript; Available in PMC 2024 Nov 1. (PMC11529804; doi:10.3389/frnar.2024.1448194)
Supplement: Supp Figure S2 [file NIHMS2027894-supplement-Supp_Figure_S2.pdf]

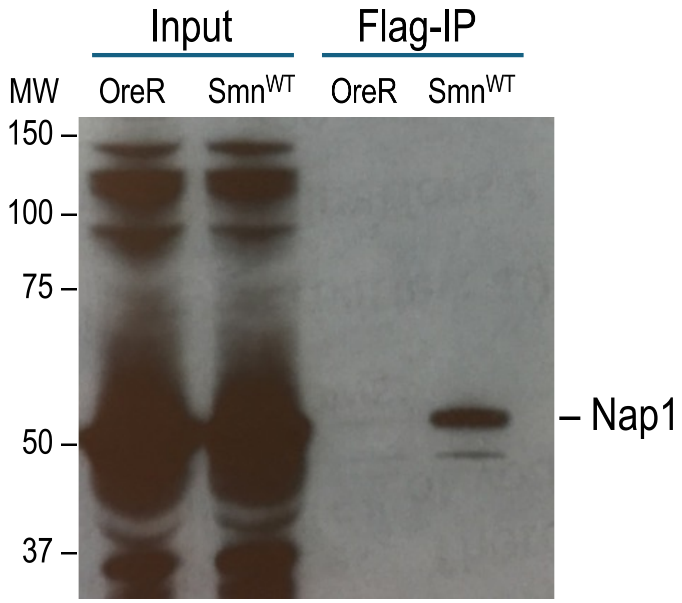

Supplementary Figure S2: Flag-purified embryonic lysates from OreR control or *Smn*^{WT} animals were probed with rabbit anti-Nap1 antibody to verify the identity of Nap1 as a protein that is co-immunoprecipitated with Flag-SMN (Flag-IP). No signal is detected in the control OreR Flag-IP, but there is a clear Nap1 band in Flag-SMN embryonic lysates. Input lanes are overexposed. Immunoprecipitation and gel image courtesy of K.M. Gray.

Fig. S2
